# Altered proTGFα/cleaved TGFα ratios offer new therapeutic strategies in renal carcinoma

**DOI:** 10.1186/s13046-021-02051-0

**Published:** 2021-08-16

**Authors:** Sara García-Alonso, Inés Romero-Pérez, Lucía Gandullo-Sánchez, Luis Chinchilla, Alberto Ocaña, Juan Carlos Montero, Atanasio Pandiella

**Affiliations:** 1 Instituto de Biología Molecular y Celular del Cáncer, CSIC and CIBERONC. Institute of Biomedical Research of Salamanca (IBSAL), Salamanca, Spain; 2 Pathology Service, University Hospital and IBSAL, Salamanca, Spain; 3 San Carlos University Hospital, Madrid, Spain

**Keywords:** Growth factors, Tyrosine kinases, EGFR, TGFα, Renal cancer

## Abstract

**Background:**

Treatment of renal cancer has significantly improved with the arrival to the clinic of kinase inhibitors and immunotherapies. However, the disease is still incurable in advanced stages. The fact that several approved inhibitors for kidney cancer target receptor tyrosine kinases (RTKs) suggests that these proteins play a critical role in the pathophysiology of the disease. Based on these precedents, we decided to explore whether RTKs other than those targeted by approved drugs, contribute to the development of kidney cancer.

**Methods:**

The activation status of 49 RTKs in 44 paired samples of normal and tumor kidney tissue was explored using antibody arrays, with validation by western blotting. Genetic and pharmacologic approaches were followed to study the biological implications of targeting the epidermal growth factor receptor (EGFR) and its ligand Transforming Growth Factor-α (TGFα).

**Results:**

Activation of the EGFR was found in a substantial number of tumors. Moreover, kidney tumors expressed elevated levels of TGFα. Down-regulation of EGFR or TGFα using RNAi or their pharmacological targeting with blocking antibodies resulted in inhibition of the proliferation of in vitro cellular models of renal cancer. Importantly, differences in the molecular forms of TGFα expressed by tumors and normal tissues were found. In fact, tumor TGFα was membrane anchored, while that expressed by normal kidney tissue was proteolytically processed.

**Conclusions:**

The EGFR-TGFα axis plays a relevant role in the pathophysiology of kidney cancer. This study unveils a distinctive feature in renal cell carcinomas, which is the presence of membrane-anchored TGFα. That characteristic could be exploited therapeutically to act on tumors expressing transmembrane TGFα, for example, with antibody drug conjugates that could recognize the extracellular region of that protein.

**Supplementary Information:**

The online version contains supplementary material available at 10.1186/s13046-021-02051-0.

## Background

Kidney cancer is one of the most common urological tumors, ranking 9th and 14th in the listing of most common tumors in men and women, respectively [1, 2]. Although the disease is still incurable in advanced stages, incorporation of immunotherapies or targeted drugs has improved patient prognosis. The targeted agents used for the therapy of kidney cancer are mainly based on kinase inhibitors such as temsirolimus or everolimus, which act on mTOR, or ample spectrum inhibitors such as sorafenib, and the receptor tyrosine kinase (RTK) inhibitors sunitinib, pazopanib, axitinib, or cabozantinib [3]. A common feature of the last four drugs is their capability to inhibit one or several of the receptors for the vascular endothelial growth factor, in addition to other RTKs [4–6]. Recently, the KEYNOTE-426 and CheckMate 9ER trials have shown that the administration of some of these RTK inhibitors with immunotherapies that act on the PD1/PD-L1 axis improves patient outcome [7, 8], underlying the important role that targeting kinases has in the therapeutics of this tumor type.

The clinical effectiveness of RTK inhibitors in kidney cancer indicates that this family of receptors plays a relevant role in the pathophysiology of the disease. Such circumstance raises the question of whether other RTKs not targeted by the drugs mentioned above may contribute to renal cancer progression. To explore such possibility, we analyzed the expression of active forms of a panel of 49 different RTKs in paired normal-tumoral tissues from patients with renal cancer. Here we report that most tumors expressed active forms of the epidermal growth factor receptor (EGFR). Moreover, we show that the tumoral samples express substantial amounts of the EGFR ligand Transforming Growth Factor-α (TGFα).

The expression and prooncogenic functions of the EGFR and TGFα in kidney cancer have already been reported [9–22], but only strategies acting on the EGFR have been tested in the clinic. Those clinical studies have reported results in different directions, with some showing lack of efficacy [23, 24] while others reporting signs of potential activity in selected populations [25, 26]. In the case of TGFα, its pathophysiological role as well as its potential manipulation with therapeutic purposes have not been explored in kidney cancer. Interestingly, *TGFA* expression has recently been linked to therapeutic responses in renal cell carcinoma [27].

The lack of information about the pathophysiological role of TGFα in kidney cancer, together with the conflicting results reported on EGFR, motivated us to further evaluate the biological relevance of targeting the EGFR-TGFα system in this pathology. We show that reduction of the expression of either EGFR or TGFα by RNAi reduced proliferation of kidney cancer cells. Moreover, the therapeutic antibody cetuximab or a neutralizing antibody against soluble TGFα reduced proliferation of cellular models of kidney cancer. These findings suggest that the EGFR-TGFα axis plays a role in the pathophysiology of kidney cancer, and therefore their targeting should be considered with therapeutic purposes. Importantly, we found differences in the molecular forms of TGFα expressed in the tumoral samples, compared to their normal paired tissues. In fact, tumoral tissues expressed a substantial amount of full length unprocessed TGFα, while normal tissues efficiently processed membrane TGFα. That finding opens the possibility of exploiting expression of membrane bound TGFα as a novel therapeutic target in kidney cancer.

## Methods

### Reagents and antibodies

Cell culture media, fetal bovine serum (FBS) and penicillin/streptomycin were from Invitrogen (Gaithersburg, MD, USA). Protein A-Sepharose was from GE Healthcare Life Sciences (Piscataway, NJ, USA). Cetuximab was from Merck (Darmstadt, Germany). Other chemicals were purchased from Sigma-Merck or Roche Biochemicals (Basel, Switzerland). The rabbit polyclonal anti-calnexin antibody was from Stressgen Biotechnologies Corporation (British Columbia, Canada). The antibody against amphiregulin was purchased from Abcam (Cambridge, UK). The neutralizing anti-human TGFα was from R&D Systems (Minneapolis, MN, USA). The antibodies against GAPDH and phosphotyrosine were from Santa Cruz Biotechnology (Santa Cruz, CA, USA). Horseradish peroxidase conjugates of anti-rabbit or anti-mouse immunoglobulin G were from Bio-Rad Laboratories (Hercules, USA). The anti-EGFR used for western blotting or immunoprecipitation and the anti-proTGFα antibodies have been described previously [28, 29].

### Patient samples and RTK phosphokinase arrays

Tyrosine phosphorylation of 49 different RTKs was evaluated by antibody arrays in renal cell carcinoma samples and normal tissues from patients of the University Hospital of Salamanca, diagnosed between 2005 and 2011. The samples used in this study were from surgical resection and were obtained following the Declaration of Helsinki on ethical principles for medical research involving human subjects. Written informed consent for research use of tissue samples was obtained from the patients.

Fresh tissue samples from the primary tumors were embedded in OCT (Thermo Fisher Scientific, Waltham, MA, USA) and then in isopentane, and placed at − 80 °C. Selection of the tumoral tissue was performed after excision of part of the tumor and staining with hematoxylin/eosin. Tissue samples were minced, washed with phosphate-buffered saline (PBS; NaCl 137 mM, KCl 2.7 mM, Na_2_HPO_4_ 8 mM, KH_2_PO_4_ 1.5 mM) and homogenized (Dispomix, L&M Biotech, Holly Springs, NC, USA) in ice-cold lysis buffer (Tris–HCl [pH 7.0] 20 mM, NaCl 140 mM, EDTA 50 mM, 10% glycerol, 1% Nonidet P-40, pepstatin 1 μM, aprotinin 1 μg/mL, leupeptin 1 μg/mL, phenylmethyl sulfonyl fluoride 1 mM, sodium orthovanadate 1 mM; 1.5 ml/100 mg of tumor). This homogenate was centrifuged at 10,000 x*g* for 20 min at 4 °C and the supernatants were transferred to new tubes.

For the antibody arrays, 300 μg of patient sample lysates were hybridized to human phospho-RTK array kit (R&D Systems), following the manufacturer’s instructions and as described [28]. Quantitation of pixel densities of the different spots corresponding to RTKs was performed using the Image Studio V5.2 program (LI-COR, Lincoln, USA).

### Cell culture, cell proliferation and infection with lentiviruses

All cell lines were cultured at 37 °C in a humidified atmosphere in the presence of 5% CO_2_ and 95% air. A-498, ACHN and Caki-2 cells were grown in Dulbecco’s Modified Eagle’s Medium (DMEM) and 769-P and 786-O in RPMI-1640 medium supplemented with 10% FBS, 100 mU/mL penicillin and 100 μg/mL streptomycin. Cell line authentication was performed by STR at the Hematology Service of the Salamanca University Hospital. Cell proliferation was assessed by cell counting [30].

Cells were seeded in 6-well plates and allowed to attach overnight in DMEM or RPMI-1640 + 10% FBS. The next day medium was replaced with complete medium containing the different antibodies. After treatment, cells were collected and counted using a Z1 Particle Counter (Beckman Coulter, Pasadena, USA). Knockdown of EGFR or TGFα in all the cell lines was performed by infection with lentiviral particles. The lentiviral vectors containing short hairpin RNA (shRNA) for EGFR or TGFα were obtained from Thermo Fisher Scientific. A minimum of 5 different shRNA sequences were tested and the ones that produced the highest knockdown levels were used for the proliferation experiments. Preparation of lentiviral vectors was performed as described previously [31].

### Immunoprecipitation and western blotting

Detailed procedures for immunoprecipitation and western have been described [32, 33]. In brief, cultured cells were washed with PBS and lysed in ice-cold lysis buffer. Lysates were centrifuged at 10,000 x*g* at 4 °C for 10 min and supernatants were transferred to new tubes with the corresponding antibody and protein A- or protein G-Sepharose. Protein concentration was determined by bicinchoninic acid (BCA) assay (Pierce BCA potein assay kit, Thermo Fisher Scientific). Immunoprecipitations were performed at 4 °C for at least 2 h. Immune complexes were recovered by a short centrifugation at 10,000 x*g* for 15 s, followed by three washes with 1 mL cold lysis buffer. Samples were then boiled in electrophoresis sample buffer and placed on SDS-PAGE gels at varying acrylamide concentrations, depending on the molecular weight of the proteins to be analyzed. After electrophoresis, the separated proteins in the gel were transferred to polyvinylidene difluoride (PVDF) membranes (Millipore Corporation, Bedford, MA, USA). Membranes were blocked in TBST (Tris [pH 7.5] 100 mM, NaCl 150 mM, 0.05% Tween 20) containing 1% BSA or 5% skimmed milk for 1–3 h and then incubated with the corresponding antibody for 2–16 h. After washing three times with TBST for 7 min, membranes were incubated with HRP-conjugated anti-mouse or anti-rabbit secondary antibodies for 30 min. After the secondary antibody, the membranes were washed three times with TBST and the bands were visualized by enhanced chemiluminescence [34].

### Quantitative PCR

Total RNA was obtained using TRIzol Reagent (Thermo Fisher Scientific). After extraction, concentration and purity were determined using a NanoDrop ND-1000 spectrophotometer (Thermo Fisher Scientific) and subsequently, 2 μg of total RNA was reverse-transcribed using MMLV-Reverse Transcriptase (Thermo Fisher Scientific) and oligodT primers in a thermocycler (Bio-Rad) under the following reaction conditions: 65 °C for 5 min, 37 °C for 50 min and 70 °C for 15 min. The cDNAs were then subjected to a real-time PCR analysis using SYBR Select Master Mix for CFX (Applied Biosystems, Foster city, CA, USA) in iQTM5 Multicolor Real Time PCR Detection System (Bio-Rad). An initial step was performed at 95 °C for 10 min, followed by 40 cycles of 95 °C for 30 s, 60 °C for 30 s and finished by 72 °C for 1 min. Each sample was analyzed in triplicate and the relative expression of each gene was determined using PP1A as housekeeping gene, according to the formula: 2^-(Ct reference gene – Ct gene). Primer sequences used were designed with Primer3 software [35] as follows:

EGF forward 5′-TCAGGGAAGATGACCACCAC-3′.

EGF reverse 5′-TCTCGGTACTGACATCGCTC-3′.

TGFA forward 5′-GAAGCCACAAAGCCGGTAAA-3′.

TGFA reverse 5′-ATACTTACCGAGGGCTCACG-3′.

AREG forward 5′-GCTGCCTTTATGTCTGCTGT-3′.

AREG reverse 5′-CACTGGAAAGAGGACCGACT-3′.

BTC forward 5′-CACCACACAATCAAAGCGGA-3′.

BTC reverse 5′-ACTCTCTCACACCTTGCTCC-3′.

HBEGF forward 5′-TGGTGCTGTCATCTGTCTGT-3′.

HBEGF reverse 5′-GTCTTTCCCCTCTGCAGTCT-3′.

EREG forward 5′-CGTGTGGCTCAAGTGTCAAT-3′.

EREG reverse 5′-TGGAACCGACGACTGTGATA-3′.

EPGN forward 5′-TGACAGCACTGACCGAAGAG-3′.

EPGN reverse 5′-CTCATGGTGGAATGCACAAG-3′.

PP1A forward 5′-ACC GCC GAG GAA AAC CGT GTA-3′.

PP1A reverse 5′-TGC TGT CTT TGG GAC CTT GTC TGC-3′.

### In silico studies

The expression analyses of EGFR and TGFα in cancer cell lines were conducted using the public genomic data available in the Cancer Cell Line Encyclopedia (CCLE) [36]. The expression analysis of EGFR and TGFα in healthy and tumor tissue samples from patient cohorts with different cancer subtypes were obtained with Firebrowse (http://firebrowse.org/) and GEPIA2 (http://gepia2.cancer-pku.cn/#general) bioinformatics tools.

### Statistical analyses

Comparisons of continuous variables between two groups were performed using a two-sided Student’s *t* test, a Wilcoxon matched-pairs signed rank test, or a Mann-Whitney U test. Differences were considered statistically significant when *p*-values were less than 0.05. Statistical data are presented as the mean ± s.d. All data were analyzed using the statistical software SPSS 21.0 (Chicago, IL, USA).

## Results

### Active RTK profiling in normal and tumoral kidney tissues

**Fig. 1:**
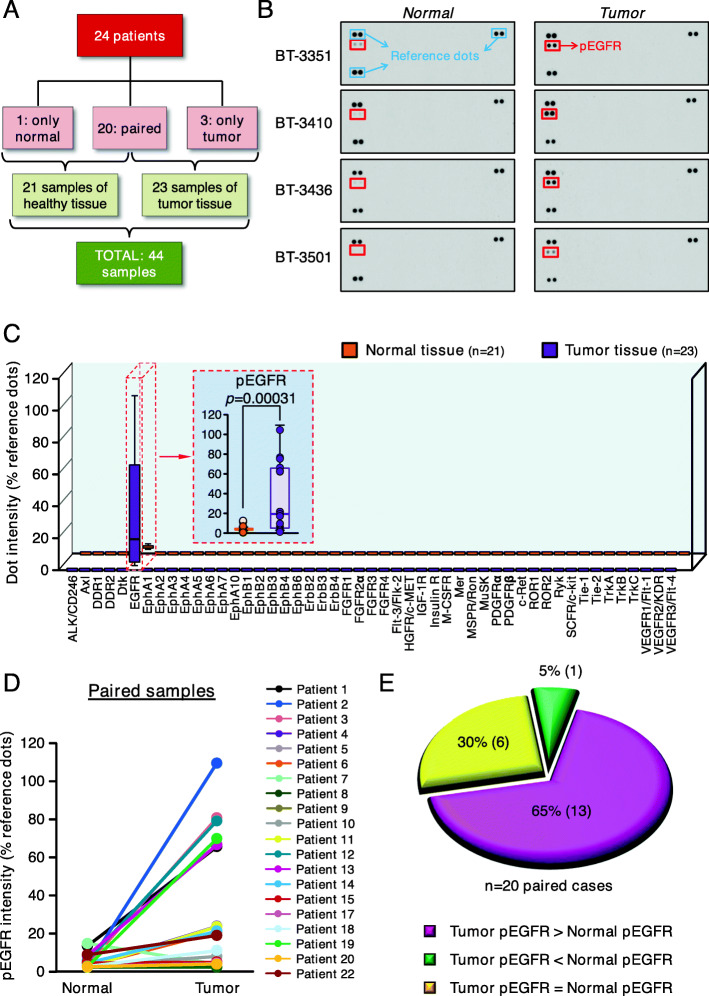
Expression of activated forms of different tyrosine kinase receptors (RTKs) in tumor and normal tissue samples from patients with renal cell carcinoma. **A**. Diagram of cases analyzed indicating the number of paired samples (tumor and healthy tissue from the same patient), as well as the total number of healthy and tumor tissue samples. **B**. Images from four representative dot blots of renal tumor samples and their normal counterparts from four different patients. **C**. Phosphorylation level of different RTKs, calculated as the mean pixel intensity of antibody duplicates relative to the reference dots. The red dashed square shows the Mann-Whitney U test comparison for EGFR activation between the normal and tumor tissue. **D**. pEGFR intensity in paired samples from 20 different renal cell carcinoma patients. **E**. Pie chart showing the percentage of paired samples where levels of pEGFR were higher, lower or equal in the tumor samples than in the healthy tissue counterpart

With the intention to find RTKs that may play a relevant pathophysiological role in the development of kidney tumors, we evaluated the activation status of 49 RTKs in kidney tissues from patients with renal cancer using antibody arrays. We preferentially searched for tumoral samples in which normal tissue from the same patient was available. That would allow comparison of RTKs activation status in the tumor with respect to the normal tissue of the same patient. Of a total of 44 samples collected from 24 patients, 40 (corresponding to 20 patients) were paired, consisting of normal and tumoral tissue from the same patient (Fig. 1A and supplementary Fig. 1A). Figure 1B shows representative arrays, corresponding to the results obtained in four normal and the corresponding tumoral paired samples. In the tumoral tissues, the level of activation of the EGFR was substantially higher than in the normal paired tissues (Fig. 1C and D). In fact, the levels of pEGFR in 13 patients (65%) were higher in the tumors than in the normal kidney tissue (Fig. 1E). In 6 patients (30%), the levels of pEGFR were similar in tumoral and normal tissue, and in only one patient pEGFR levels were higher in the normal kidney than in the tumoral tissue. No tyrosine phosphorylation of the other 48 RTKs analyzed was detected.

Immunoprecipitation studies were carried out on all paired cases to confirm the activation of EGFR in the tumoral samples. These studies confirmed much higher pEGFR in tumoral samples as compared to the paired normal kidney tissue (supplementary Fig. 1B). Moreover, the amount of total EGFR in those tumoral samples was also superior to the amount present in the normal counterparts, suggesting that not only increased levels of pEGFR were present in the tumoral samples, but also elevated levels of the receptor.

Next, the expression of EGFR in different tumors was explored using Firebrowse and GEPIA2 databases, which also allow comparison of expression levels between normal and tumoral tissue. Analyses of the former database showed that kidney tumors ranked the second type of cancer with the highest EGFR expression of the 19 available for analysis (supplementary Fig. 2A). In the case of the GEPIA2 database, kidney cancer ranked third of 33 different tumor types, with respect to EGFR amount (supplementary Fig. 2B). In both databases, EGFR expression was higher in renal tumoral tissue than in normal kidney, in line with data from the five paired samples presented in supplementary Fig. 1B.

### EGFR ligands in normal and tumoral kidney tissues

**Fig. 2:**
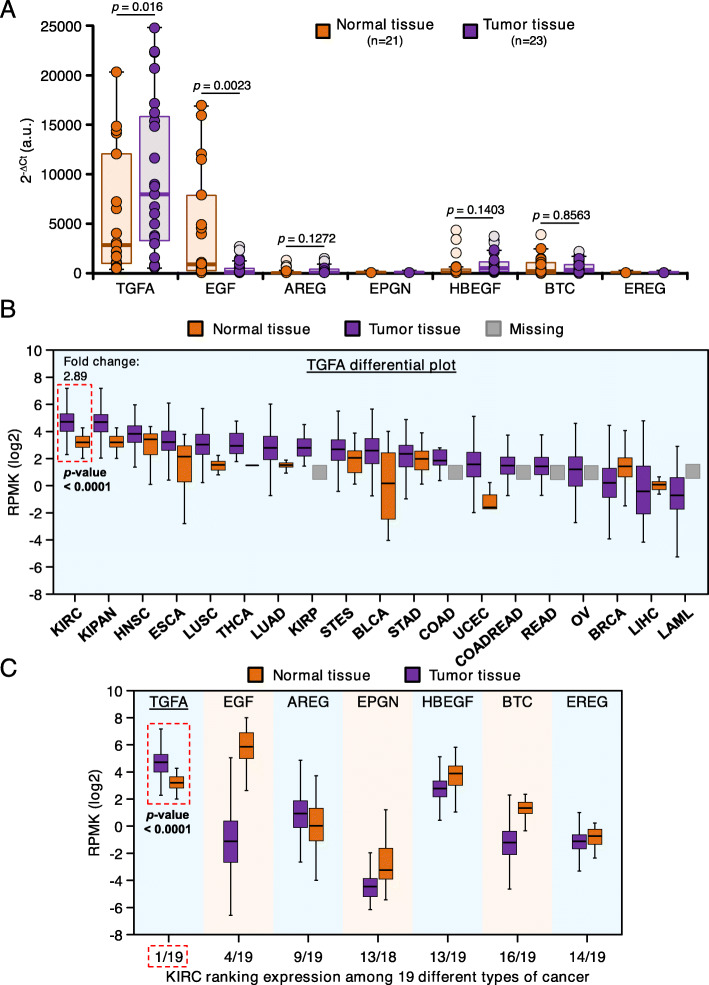
Differential expression of EGFR ligands in healthy and tumor tissue. **A**. Level of expression (measured by quantitative PCR) of the seven EGFR ligands in normal (*n* = 21, orange) and tumor (*n* = 23, purple) samples from renal cell carcinoma patients. Data are represented as 2^-delta Ct, in arbitrary units (a.u.). *P* values are indicated. **B**. Differential expression of TGFA in 19 cancer subtypes, both in normal and tumor tissue. RNA-Seq data were obtained from Firebrowse database and are represented as RPKM (reads per kilobase of exon model per million mapped reads). The expression of TGFA in kidney renal clear cell carcinoma is highlighted with a red dashed square, indicating the expression fold change between tumor and normal tissue. KIRC, kidney renal clear cell carcinoma; KIPAN, pan-kidney cohort; HNSC, head and neck squamous cell carcinoma; ESCA, esophageal carcinoma; LUSC, lung squamous cell carcinoma; THCA, thyroid carcinoma; LUAD, lung adenocarcinoma; KIRP, kidney renal papillary cell carcinoma; STES, stomach and esophageal carcinoma; BLCA, bladder urothelial carcinoma; STAD, stomach adenocarcinoma; COAD, colon adenocarcinoma; UCEC, uterine corpus endometrial carcinoma; COADREAD, colorectal adenocarcinoma; READ, rectum adenocarcinoma; OV, ovarian serous cystadenocarcinoma; BRCA, breast invasive carcinoma; LIHC, liver hepatocellular carcinoma; LAML, acute myeloid leukemia. **C**. Differential expression of the seven EGFR ligands in kidney renal clear cell carcinoma and normal tissue. The plot shows the rank of this cancer subtype among the 19 analyzed ones in terms of ligand expression

Since the EGFR appeared highly phosphorylated, the expression of its seven ligands was then explored. Quantitative PCR analyses showed expression of EGF and TGFα in the normal tissues (Fig. 2A). In addition, TGFα expression was high in the tumoral samples. In contrast, the levels of EGF in tumoral samples were lower than in the normal tissue. Expression of the other EGFR ligands amphiregulin, betacellulin, epigen, HB-EGF or epiregulin was very low in both normal and neoplastic kidney tissues.

Exploration of Firebrowse (Fig. 2B) and GEPIA2 (supplementary Fig. 3A) databases showed that kidney cancer ranked first with respect to TGFα expression when compared to other tumoral types represented in those databases. In agreement with the data obtained from the samples analyzed in Fig. 2A, the in silico data confirmed that TGFα expression was higher in tumoral tissue than in normal kidney (Fig. 2B and supplementary Fig. 3B). Analysis of Firebrowse database confirmed TGFα as the most abundantly expressed of the seven EGFR ligands in the tumoral samples (Fig. 2C). Moreover, with the exception of amphiregulin, the EGFR ligands appeared to be more expressed in normal samples than in tumoral ones. Of the tumors present in this database, kidney cancer ranked first with respect to TGFα expression levels. Together, these observations indicate that among all EGFR ligands, TGFα is the only one expressed at high levels in kidney cancer.

### TGFα molecular forms in normal and tumoral kidney tissues

**Fig. 3:**
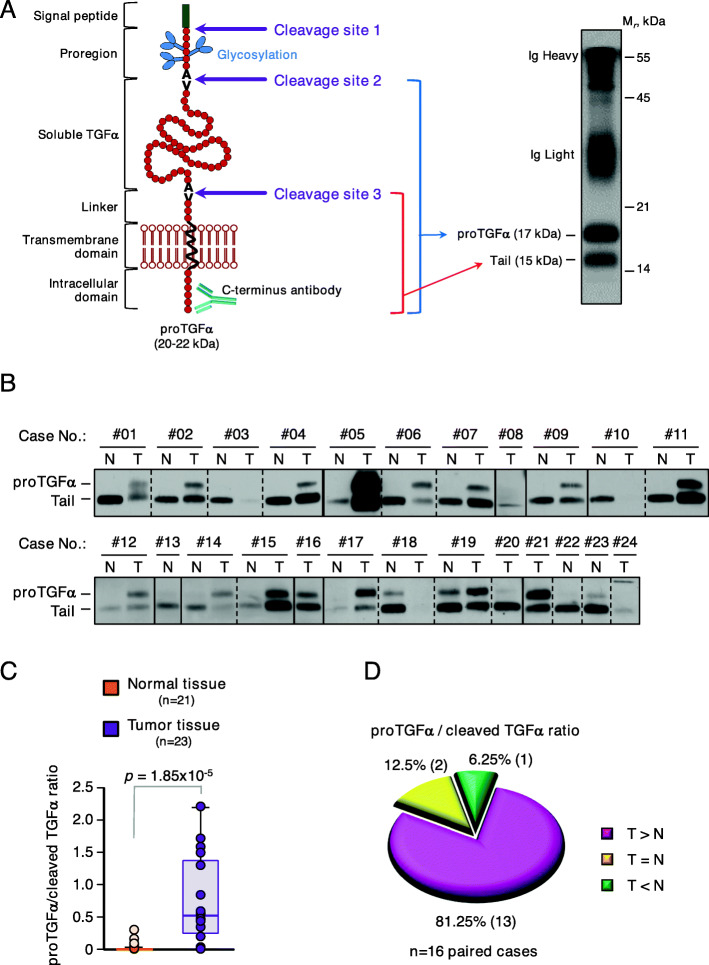
TGFα protein levels in renal cancer patients. **A**. (Left) Schematic representation of proTGFα structure, indicating domains and the proteolytic cleavage sites that lead to the membrane anchored proTGFα or tail. The antibody used for western blotting detection is showed as well. (Right) Western blot showing the different forms of TGFα detected in a renal cell carcinoma sample. **B**. Protein levels of different molecular forms of TGFα in normal and tumor kidney tissues from 24 patients. The same antibody was used for both the immunoprecipitation and the western blot. **C**. Ratio of proTGFα and tail fragment between tumor and normal tissue, calculated as the mean pixel intensity of the upper band (17 KDa) to the lower band (15 KDa) of the above WB scans. *p*-value was calculated by the Mann-Whitney U test. **D**. Pie chart showing the percentage of paired samples where levels of TGFα were higher (pink), lower (green) or equal (yellow) in the tumor samples than in the healthy tissue counterpart

TGFα is biosynthesized as a transmembrane precursor protein, known as proTGFα, which undergoes three proteolytic cleavages (Fig. 3A). Cleavage at site 1 removes the N-terminal signal peptide and is expected to occur co-translationally [29, 37, 38]. Then, the protein undergoes maturation along the Golgi complex to generate 20–22 heterogeneously glycosylated forms that are cleaved at site 2 to generate a transmembrane 17 kDa proTGFα form [37, 39]. These two cleavage steps occur relatively rapid during the initial steps of proTGFα synthesis and maturation [29]. In contrast, the 17 kDa proTGFα form may accumulate at the cell surface and is processed under circumstances, such as activation of certain kinase routes that stimulate the activity of proTGFα secretases [29, 33, 40]. The action of these secretases on proTGFα results in release of soluble TGFα together with the generation of a cell-bound tail fragment of 15 kDa. Preliminary western blotting studies of a renal tumoral tissue sample, using an antibody raised to the C-terminal intracellular region of proTGFα, showed that the specific sample shown in Fig. 3A mainly contained the 17 and 15 kDa forms. This antibody was used to explore the prevalence of the different molecular forms of TGFα in normal and tumoral kidney tissues. Most normal kidney tissue samples predominantly expressed the 15 kDa form (Fig. 3B). Moreover, expression of the 17 kDa form could not easily be detected in most of the samples, indicating that processing of the 17 to the 15 kDa form was efficient in the normal kidney tissues. In contrast, expression of the 17 kDa form was detected in most tumoral samples, in addition to the 15 kDa form. Quantitative analysis of the 17/15 kDa band ratio showed significant differences (*p* = 1.85 × 10^− 5^) between normal tissues with respect to the tumoral ones (Fig. 3C). Specifically, in 13 of the analyzed paired samples (81.25%) the 17/15 kDa ratio was higher in the tumoral tissue than in the normal paired tissue, while those ratios were similar in 2 paired samples (12.5%, Fig. 3D). Lower ratio of the 17/15 kDa forms in the tumor with respect to the normal tissue was only seen in 1 patient (6.25%).

### Expression and role of EGFR and TGFα in the proliferation of kidney cancer cell lines

**Fig. 4:**
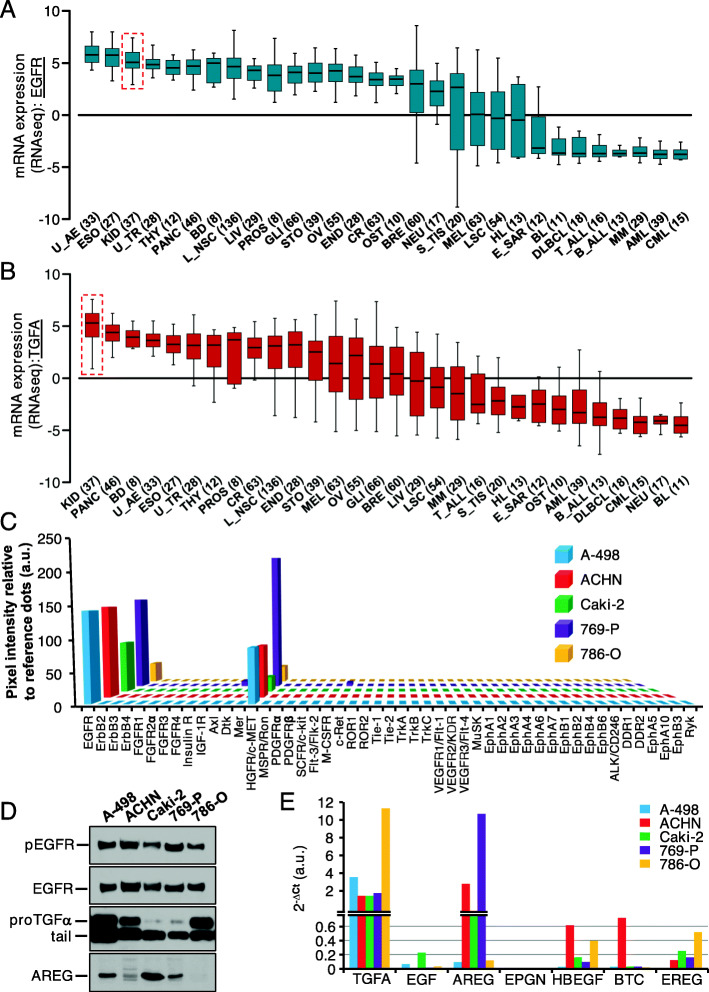
Expression of EGFR and TGFα in kidney cancer cell lines. **A**. Differential expression of EGFR and **B**. TGFA in different cancer cell lines. RNA-Seq data were obtained from the Cancer Cell Line Encyclopedia. The number of different cell lines for each cancer subtype is indicated in brackets and the data corresponding to the expression of both genes in kidney are highlighted with a red dashed square. **C**. Phosphorylation level of different RTKs, calculated as the mean pixel intensity of dot duplicates from the antibody array relative to the reference dots (in a.u.). **D**. Protein levels of phosphorylated and total EGFR, as well as two ligands (TGFα and amphiregullin) in kidney cancer cell lines. **E**. Level of expression (measured by quantitative PCR) of the seven EGFR ligands in the cell lines. Data are represented as 2^-delta Ct, in arbitrary units (a.u.). U_AE, upper aerodigestive; ESO, esophagus; KID, kidney; U_TR, urinary tract; THY, thyroid; PANC, pancreas; BD, bile duct; L_NSC, lung non-small cell carcinoma; LIV, liver; PROS, prostate; GLI, glioma; STO, stomach; OV, ovary; END, endometrium; CR, colorectal; OST, osteosarcoma; BRE, breast; NEU, neuroblastoma; S_TIS, soft tissue; MEL, melanoma; LSC, lung small cells; HL, Hodgkin lymphoma; E_SAR, Ewing sarcoma; BL, Burkitt lymphoma; DLBCL, diffuse large B-cell lymphoma; T_ALL, T-cell acute lymphoid leukemia; B_ALL, B-cell acute lymphoid leukemia; MM, multiple myeloma; AML, acute myeloid leukemia; CML, chronic myeloid leukemia

To functionally explore the role of the EGFR/TGFα system in kidney cancer, several established human cell lines were selected. To help in that purpose in silico available data from the cancer cell line encyclopedia [41] was first evaluated. Data obtained from that database confirmed that the EGFR was highly expressed in kidney cancer cell lines, quantitatively ranking as the third tumoral type, behind upper aerodigestive tract and esophagus (Fig. 4A). Similar analyses showed that kidney cancer ranked first with respect to TGFα expression (Fig. 4B). Comparative RNAseq data of the 37 kidney cancer cell lines represented in such database showed expected variability among their *EGFR* and *TGFA* mRNA levels and copy number (supplementary Fig. 4A and B). On the bases of these data, we selected two cell lines (ACHN and A-498) representative of the highest (> 0.3) TGFA copy number alterations, one cell line (769-P) within the lowest range (0–0.1), and two cell lines (786-O and Caki-2) with intermediate (0.1–0.3 0.3) copy number alterations. Levels of *EGFR* copy number alterations qualitatively paralleled those of *TGFA* (ACHN > A-498 > Caki-2 > 786-O > 769-P).

Antibody arrays and western blotting demonstrated that the EGFR was phosphorylated in the five cell lines analyzed (Fig. 4C and D and supplementary Fig. 5). In addition, these studies also showed activation of the HGFR/c-MET receptor. Western blotting analyses showed that the levels of total or phosphorylated EGFR were similar in the five cell lines analyzed (Fig. 4D), in agreement with the data obtained by the RNAseq analysis available from the cancer cell line encyclopedia shown in supplementary Fig. 4A.

With respect to the EGFR ligands, qPCR analyses showed expression of TGFα and amphiregulin (Fig. 4E). HB-EGF, betacellulin and epiregulin were expressed at lower levels, while EGF and epigen were almost undetectable. Western blotting confirmed expression of TGFα in the five cell lines analyzed and amphiregulin in four of five (Fig. 4D). The pattern of expression of the different molecular forms of proTGFα varied among the five cell lines studied. In A-498, ACHN and 786-O cells, the 17 kDa proTGFα form was clearly detectable (Fig. 4D). In contrast, in Caki-2 and 769-P cells such 17 kDa form was low in amount and in some experiments difficult to detect, particularly in Caki-2 cells.

**Fig. 5:**
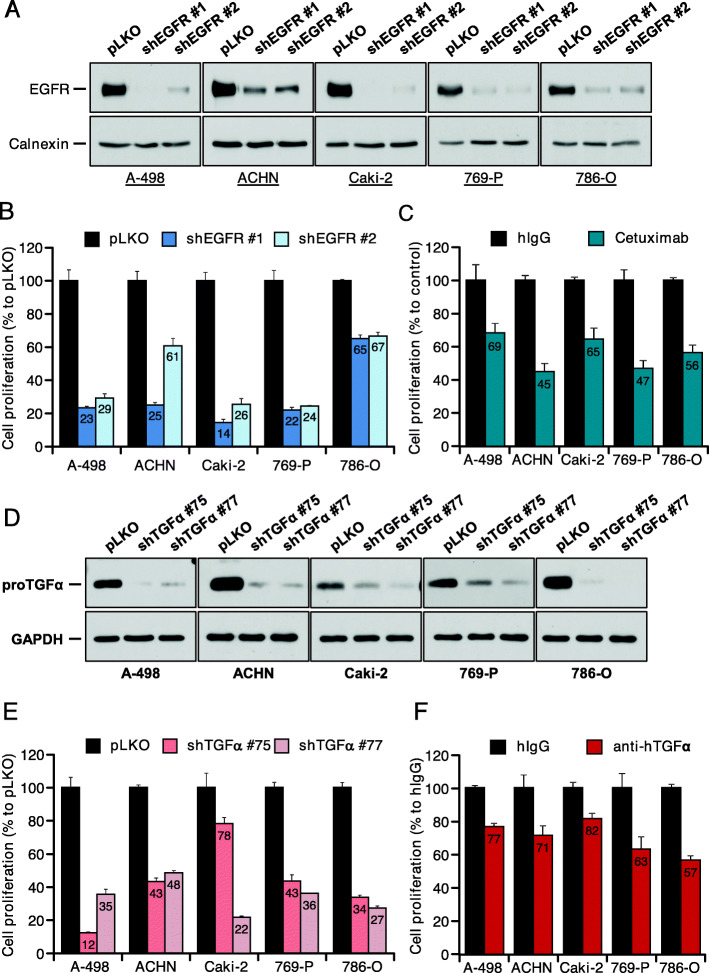
Role of EGFR and TGFα in the proliferation of kidney cancer cell lines. **A**. Knockdown of EGFR and **D**. TGFα in renal cancer cell lines. Cells were infected with control vector (pLKO) and viruses including two different short hairpin sequences targeting EGFR (**A**) and TGFA (**D**). Cell extracts were obtained and the receptor expression was measured by immunoprecipitation followed by western blot with the specific antibody. **B**. EGFR or **E**. TGFα knockdown effect on the proliferation of renal cancer cells. Cells were infected with the indicated shRNAs. After selection, cells were then plated and counted after 5 days. Results are plotted as the mean ± s.d. of triplicates with respect to the proliferation of cultures infected with the control vector. **C**. Effect of cetuximab on the proliferation of renal cancer cells. Cells were treated with 10 nM of cetuximab or anti-human IgG antibody (hIgG) for 5 days and then counted. Graph bars represent the mean ± s.d. of triplicates normalized to cells treated with hIgG as a control. **F**. Effect of a neutralizing anti-TGFα antibody on the proliferation of renal cancer cells. Cells were treated as in **C**. and counted

To explore whether the TGFα/EGFR axis could play a role in the pathophysiology of kidney cancer, we used genetic and pharmacologic approaches to explore the effect of targeting the EGFR on the proliferation of kidney cancer cells. The genetic approach was based on RNAi of EGFR. Five different short hairpin lentiviral sequences were analyzed for their capability to decrease expression of EGFR. Of these sequences we selected two of them that caused substantial decrease in the EGFR in the five cell lines (Fig. 5A). The two shRNA sequences decreased the proliferation of these cells (Fig. 5B). The effect of pharmacological targeting the EGFR on cell proliferation was analyzed by using the clinical drug cetuximab, which interacts with the extracellular region of the EGFR [42]. Treatment with this antibody decreased the proliferation of all five kidney cancer cell lines (Fig. 5C).

Next, and by using similar approaches, the impact of TGFα targeting on the proliferation of kidney cancer cells was studied. The effect of the five RNAi sequences initially used on the levels of 17 kDa proTGFα is shown in Fig. 5D. These experiments allowed selection of two shRNA sequences (#75 and #77) which efficiently decreased the levels of the factor in all five cell lines (Fig. 5D). Both sequences substantially affected the proliferation of the five different cell lines (Fig. 5E). Finally, the effect of a neutralizing anti-TGFα antibody was also tested. Treatment of the five cell lines with this antibody decreased their proliferation (Fig. 5F).

## Discussion

Despite significant advances in the therapy of kidney cancer, a proportion of patients cannot be cured by surgical or pharmacological treatment [3, 6]. Therefore, the finding of new pathophysiological actors in kidney cancer could help in fighting this disease. The present study was initiated with the purpose of increasing our knowledge about the contribution of RTKs to the pathophysiology of kidney cancer, considering the clinical benefit obtained in kidney cancer by multitarget tyrosine kinase inhibitors as sunitinib or pazopanib [3].

In our study design, special attention was given to the selection of samples. It was considered important to look for tumors from which normal tissue would also be available. That would allow the comparison of the RTKs activation status between the normal and the tumoral tissues of the same patient, and this was expected to give a more accurate view of the potential participation of the analyzed RTKs in kidney cancer. These studies demonstrated that the EGFR was active (tyrosine phosphorylated) in a substantial number of the tumoral samples. In contrast, tyrosine phosphorylation of 48 other RTKs analyzed was inappreciable. Unexpectedly, we did not find activation of RTKs that act as major targets of the clinically approved drugs. In contrast, activation of the EGFR was found in a substantial number of tumors. In fact, in thirteen of the twenty paired tumor-normal samples studied, the tumoral sample expressed higher levels of pEGFR than the normal tissue. The inverse situation, i.e., higher pEGFR in normal tissue than in tumoral samples, was observed in only one case. Given the fact that targeting EGFR has demonstrated therapeutic benefit in other neoplastic diseases [43], our results support the possibility of acting on this receptor. Such idea should be considered in the context of former clinical studies using agents that target the EGFR. While the EGFR tyrosine kinase inhibitor gefitinib did not offer clinical benefit in a small clinical trial [23]; in the case of lapatinib, a randomized clinical study showed potential signs of activity particularly in patients with high expression of the receptor [25]. In the case of antibodies targeting the EGFR, a study using cetuximab in a limited number of patients demonstrated lack of activity [24], while Rowinsky et al. showed that ABX-EGF, another humanized anti-EGFR antibody exerted some clinical benefit [26]. This mixed information, with some reports showing lack of activity, and others signs of potential benefit, decreased the interest of pharmaceutical sponsors to promote the development of anti-EGFR strategies for renal cancer. On the other hand, that high grade of uncertainty motivated us to preclinically explore the role of EGFR and TGFα in kidney cancer, as they could be used as druggable targets. In fact, the cell proliferation data obtained by genetic as well as the pharmacologic targeting of EGFR and TGFα suggest that acting on this system may be used therapeutically in kidney cancer.

The analyses of the paired samples showed striking differences in the molecular forms of proTGFα in normal versus tumoral samples. Normal kidney tissues mainly expressed processed proTGFα, indicated by accumulation of the 15 kDa tail form. In the tumoral samples, and in addition to the 15 kDa form, the 17 kDa proTGFα form was also present. Our unpublished data indicated that those different ratios could not be attributed to significantly different levels of expression of the transmembrane metalloprotease TACE, one of the major proTGFα processing proteases [44]. The lack of major changes in TACE expression between normal and tumoral kidney tissues suggests that differences in the molecular forms of proTGFα present in those tissues must depend on other factors, such as the degree of activation of TACE [45] or alternative proTGFα secretases [33, 44]. Petrides et al. published that tumoral kidney tissues express a 4.8 kb *TGFA* transcript that is not found in normal kidney tissue [46]. That finding opens the possibility that such 4.8 kb form may contribute to the different proTGFα/TGFα ratios. However, the lack of published data in that direction, together with the well-known posttranscriptional source of the 17 and 15 kDa forms suggests that the forms present in the kidney samples derive from the synthesis and maturation of a common TGFA transcript [29]. While the functional consequences of such differences in the expression of the various proTGFα molecular forms require further analyses, it is relevant to mention that transmembrane forms of membrane-anchored growth factors may be active [47, 48]. In the normal tissue, cleavage of proTGFα should result in the generation of soluble TGFα, which can then freely navigate across the extracellular spaces. That circumstance may favor disappearance of soluble TGFα from the kidney tissue either by diffusion to the bloodstream, or by internalization and degradation by EGFR expressed by normal parenchymatous kidney cells. In any case, the absence of soluble TGFα should restrict activation of the EGFR in normal kidney tissue. On the other hand, uncleaved proTGFα can reside at the cell surface and therefore remain accessible to EGFR of neighboring tumoral cells. That circumstance may favor the proliferation of kidney tumors expressing uncleaved proTGFα and its receptor.

While targeting TGFα with antibodies that block its interaction with the EGFR may inhibit tumor progression [49], our findings on the molecular forms of TGFα expressed in kidney tumors open a new therapeutic scenario. The presence of uncleaved proTGFα in the diseased tumoral tissue offers the interesting possibility of directing antibody-drug conjugates (ADC) to the tumoral cells expressing transmembrane proTGFα. This is an attractive hypothesis that could be tested upon availability of such antibody derivatives. Such idea also implicates the presence of sufficient proTGFα at the cell surface, as well as assuming that the unclipped membrane-bound factor would internalize upon binding to the ADC. Some studies have in fact indicated that proTGFα can internalize [50], opening the possibility that this membrane-anchored growth factor may be used as an ADC target. Future studies should uncover whether proTGFα and other membrane-anchored growth factors of the EGF family are suitable ADC targets.

## Conclusions

Analyses of paired normal-tumoral patient samples showed that active EGFR and TGFα are frequently expressed in kidney cancer. Moreover, pharmacological as well as genetic manipulations demonstrated the potential therapeutic value of targeting this receptor-ligand pair in that disease. Moreover, biochemical characterization of TGFα showed important differences between the molecular forms of TGFα expressed in normal and tumoral kidney tissues. We propose that such differences could be therapeutically exploited.

## Supplementary Information

**Additional file 1: Supplementary Fig. 1. A.** Data from patient samples used in the study (case number, histopathology database code and availability of paired samples). **B.** EGFR phosphorylation levels in renal cancer patients. Protein lysates were immunoprecipitated with an anti-EGFR antibody and the activation status was detected by western blotting using an anti-phosphotyrosine antibody. Total levels of EGFR were directly analyzed on cell extracts. Examples from 5 different patients are shown.

**Additional file 2: Supplementary Fig. 2. A**. Expression of EGFR in normal and tumor tissue. **A.** Data obtained from the Firebrowse database, corresponding to 19 cancer subtypes. **B.** Data obtained from the GEPIA2 database, corresponding to 33 different tumor types. RNA-Seq data in part A are represented as in Fig. 2B.

**Additional file 3: Supplementary Fig. 3.** Expression of TGFA in normal and tumor tissue. **A.** TGFA expression in 31 normal and tumoral tissues, obtained using the GEPIA2 database. **B.** Specific analysis of the expression of TGFA in tumoral vs. normal kidney tissue, showing that expression in the tumoral tissue is significantly higher than in normal tissue.

**Additional file 4: Supplementary Fig. 4.** Expression and copy number data of EGFR (A) and TGFA (B) in cells represented in the Cancer Cell Line Encyclopedia. The positions of the cells selected for the study herewith presented are indicated.

**Additional file 5: Supplementary Fig. 5.** RTK activation arrays of renal cancer cell lines. The activated RTKs detected are highlighted with a red square.

## Data Availability

The online datasets analysed during the current study are available in the Cancer Cell Line Encyclopedia (CCLE), Firebrowse (http://firebrowse.org/) and GEPIA2 (http://gepia2.cancer-pku.cn/#general) repositories. All data generated from patient frozen samples during the current study are available in the present paper and additional data may be available by contacting the corresponding author.

## Abbreviations

BCA: Bicinchoninic acid
CCLE: Cancer Cell Line Encyclopedia
DMEM: Dulbecco’s Modified Eagle’s Medium
EGF: Epidermal growth factor
EGFR: Epidermal growth factor receptor
FBS: Fetal bovine serum
PVDF: Polyvinylidene difluoride
RTK: Receptor tyrosine kinase
shRNA: Short hairpin RNA
TGFα: Transforming growth factor-α
