# Supplementary material for: Altered proTGFα/cleaved TGFα ratios offer new therapeutic strategies in renal carcinoma
Source: J Exp Clin Cancer Res. 2021 Aug 16;40:256. doi: 10.1186/s13046-021-02051-0 (PMC8365933; doi:10.1186/s13046-021-02051-0)
Supplement: Supplementary file 1 — Additional file 1: Supplementary Fig. 1. A. Data from patient samples used in the study (case number, histopathology database code and availability of paired samples). B. EGFR phosphorylation levels in renal cancer patients. Protein lysates were immunoprecipitated with an anti-EGFR antibody and the activation status was detected by western blotting using an anti-phosphotyrosine antibody. Total levels of EGFR were directly analyzed on cell extracts. Examples from 5 different patients are shown. [file 13046_2021_2051_MOESM1_ESM.pdf]

A

Patient 01	BT-3351	Paired
Patient 02	BT-3410	Paired
Patient 03	BT-3436	Paired
Patient 04	BT-3501	Paired
Patient 05	BT-3514	Paired
Patient 06	BT-3521	Paired
Patient 07	BT-3536	Paired
Patient 08	BT-3700	Paired
Patient 09	BT-3731	Paired
Patient 10	BT-3775	Paired
Patient 11	BT-3776	Paired
Patient 12	BT-3116	Paired
Patient 13	BT-3124	Paired
Patient 14	BT-3246	Paired
Patient 15	BT-3261	Paired
Patient 16	BT-3268	Tumor only
Patient 17	BT-3115	Paired
Patient 18	BT-3041	Paired
Patient 19	BT-3036	Paired
Patient 20	BT-3033	Paired
Patient 21	BT-3056	Tumor only
Patient 22	BT-2986	Paired
Patient 23	BT-2999	Healthy tissue
Patient 24	BT-2957	Tumor only

B

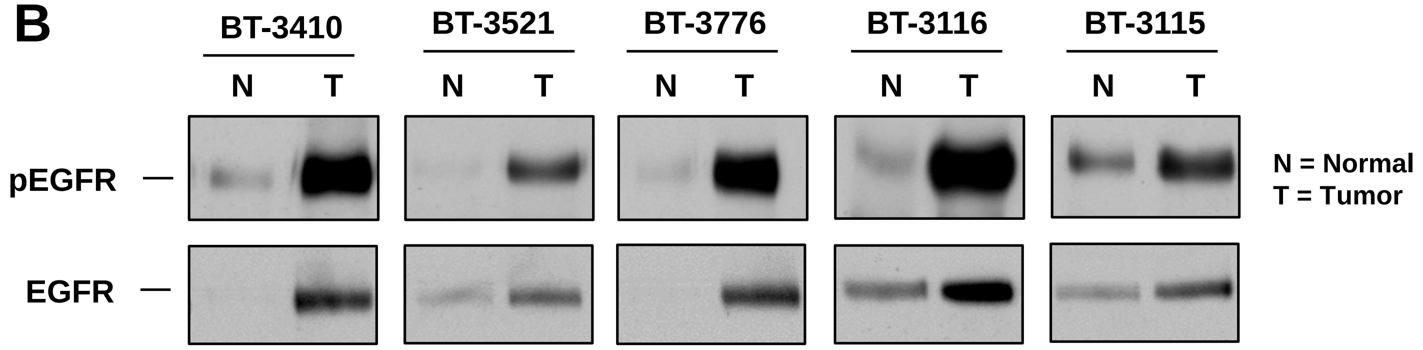

Supplementary figure 1
