# Supplementary figures and images for: Altered proTGFα/cleaved TGFα ratios offer new therapeutic strategies in renal carcinoma
Source: J Exp Clin Cancer Res. 2021 Aug 16;40:256. doi: 10.1186/s13046-021-02051-0 (PMC8365933; doi:10.1186/s13046-021-02051-0)

**A**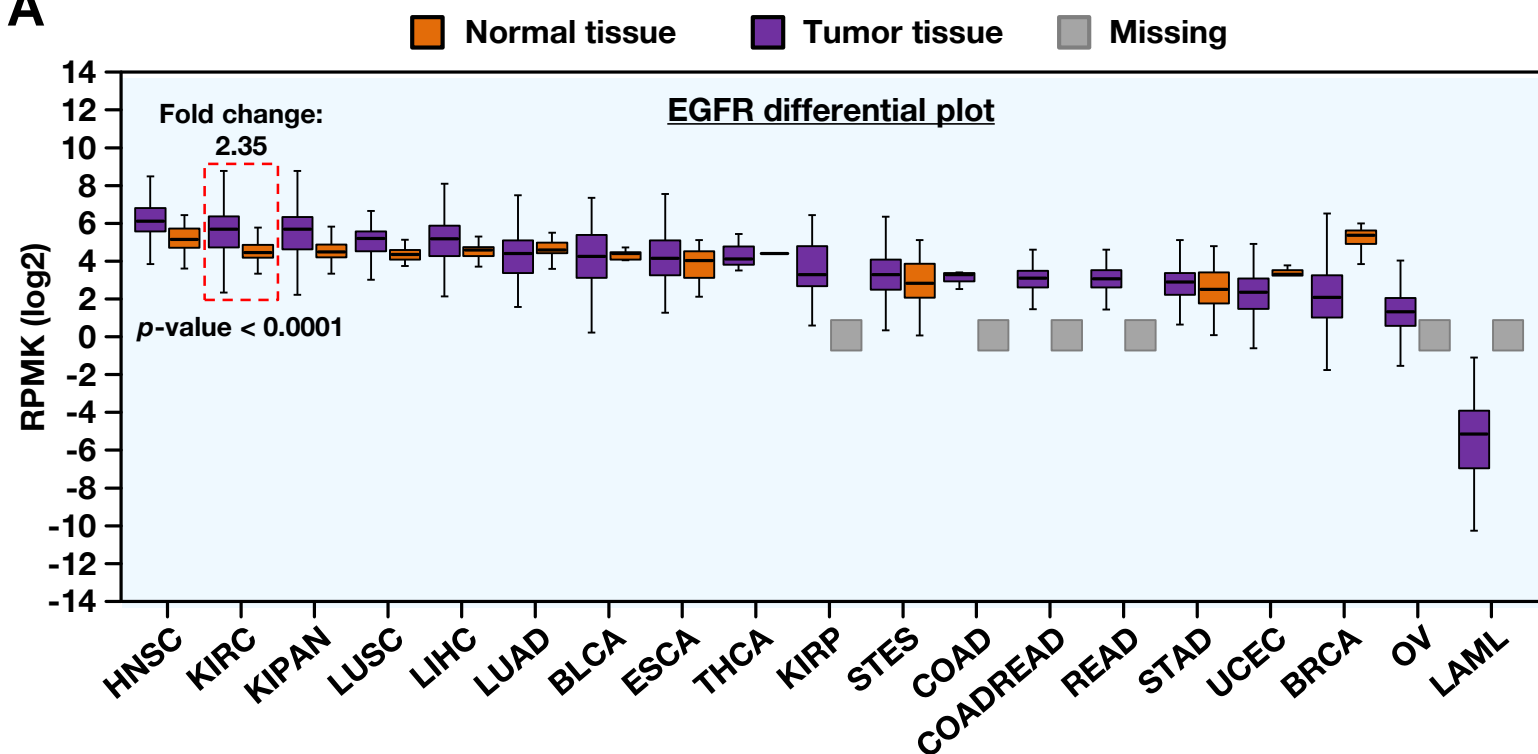**B**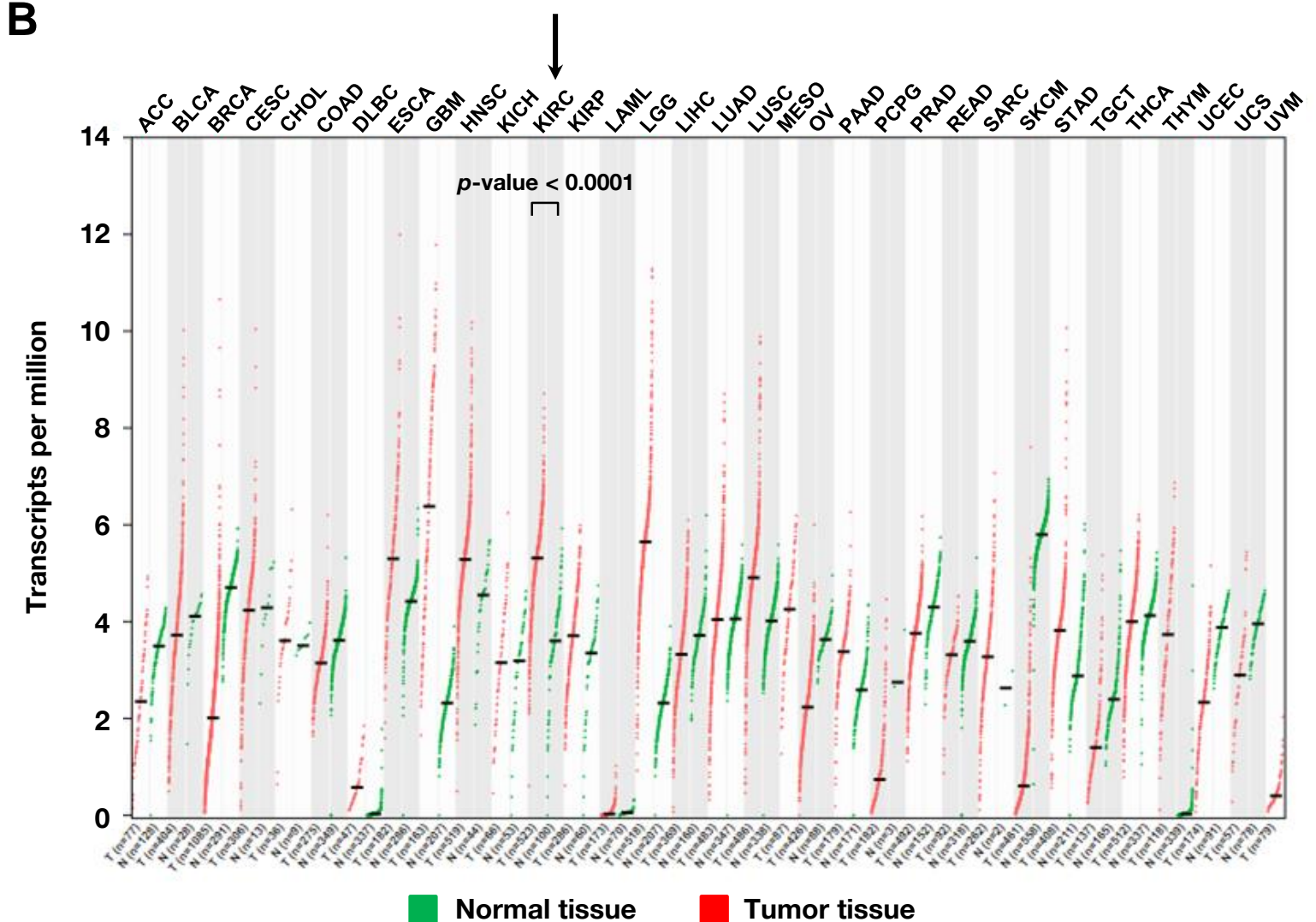**Supplementary figure 2**

Supplement: Supplementary file 2 — Additional file 2: Supplementary Fig. 2. A. Expression of EGFR in normal and tumor tissue. A. Data obtained from the Firebrowse database, corresponding to 19 cancer subtypes. B. Data obtained from the GEPIA2 database, corresponding to 33 different tumor types. RNA-Seq data in part A are represented as in Fig. 2B. [file 13046_2021_2051_MOESM2_ESM.pdf]

**A**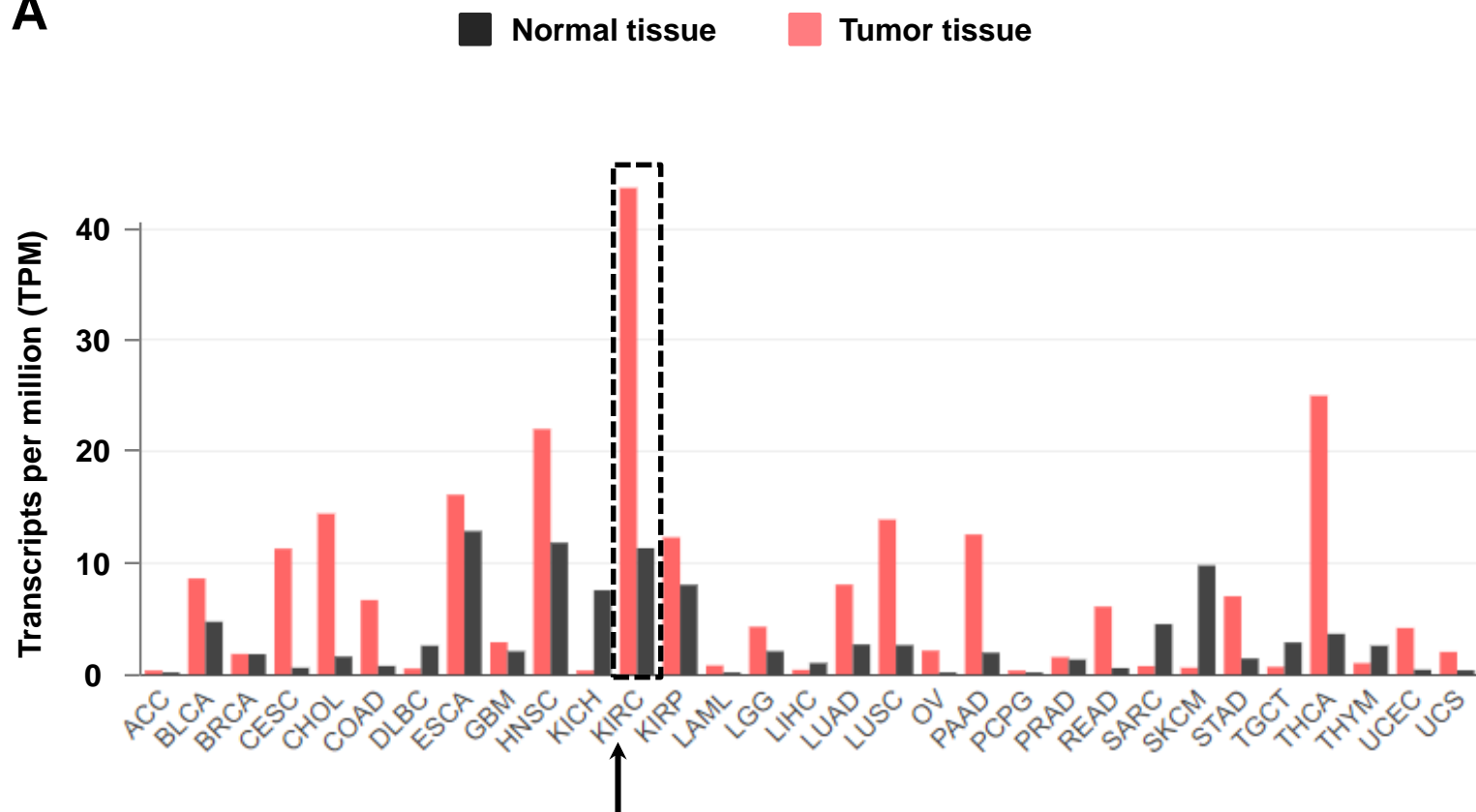**B**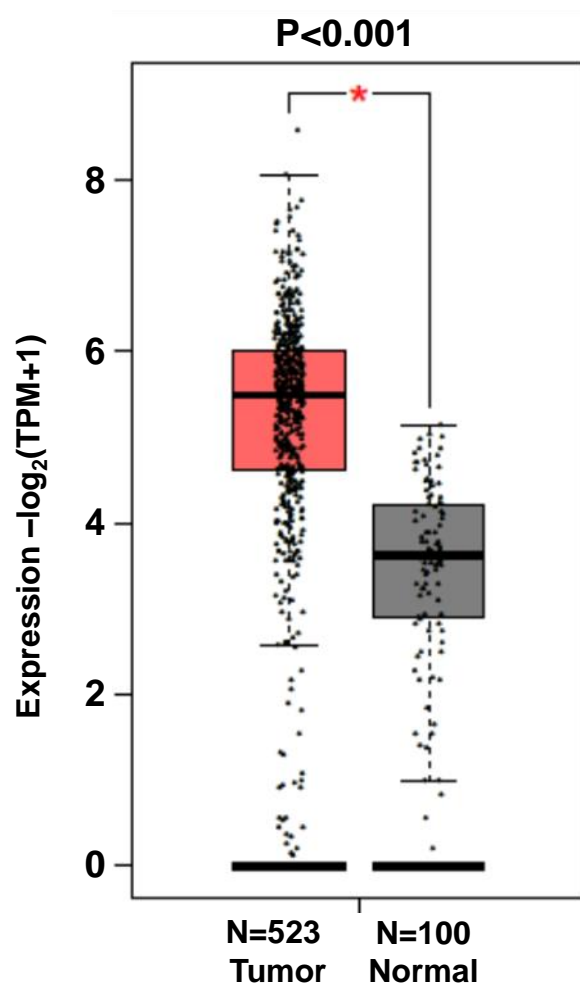**Supplementary figure 3**

Supplement: Supplementary file 3 — Additional file 3: Supplementary Fig. 3. Expression of TGFA in normal and tumor tissue. A. TGFA expression in 31 normal and tumoral tissues, obtained using the GEPIA2 database. B. Specific analysis of the expression of TGFA in tumoral vs. normal kidney tissue, showing that expression in the tumoral tissue is significantly higher than in normal tissue. [file 13046_2021_2051_MOESM3_ESM.pdf]

**A**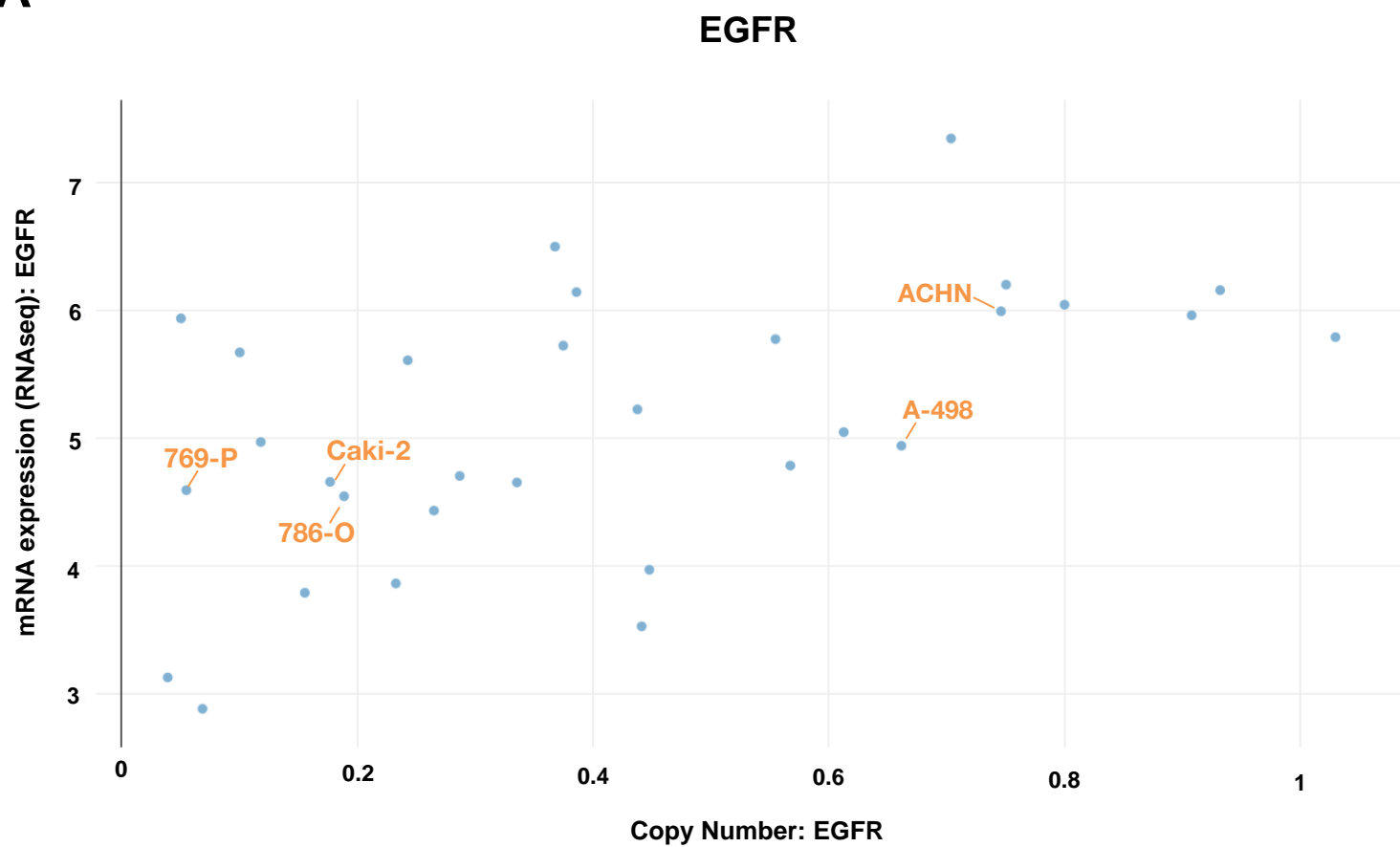**B**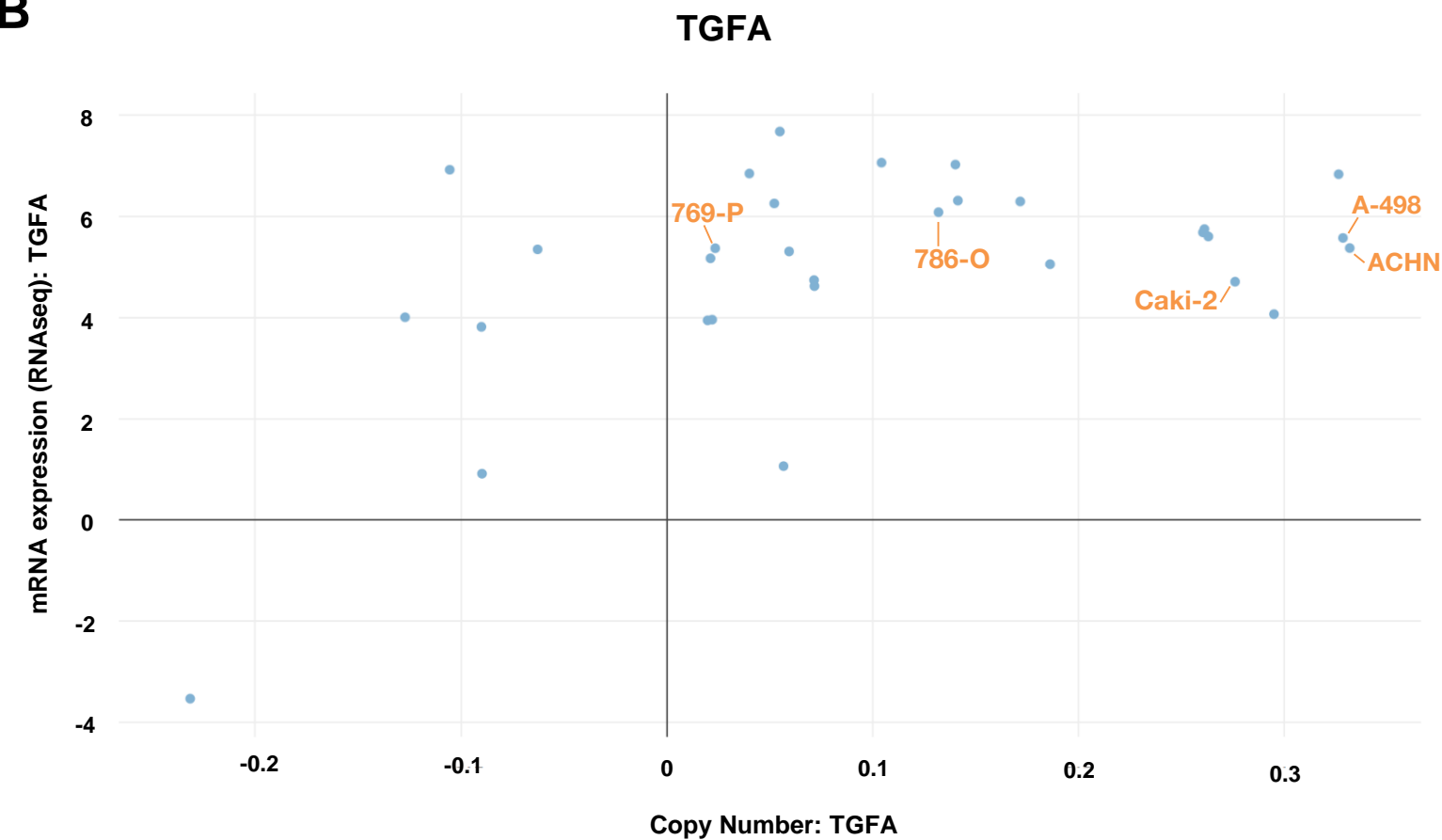**Supplementary figure 4**

Supplement: Supplementary file 4 — Additional file 4: Supplementary Fig. 4. Expression and copy number data of EGFR (A) and TGFA (B) in cells represented in the Cancer Cell Line Encyclopedia. The positions of the cells selected for the study herewith presented are indicated. [file 13046_2021_2051_MOESM4_ESM.pdf]

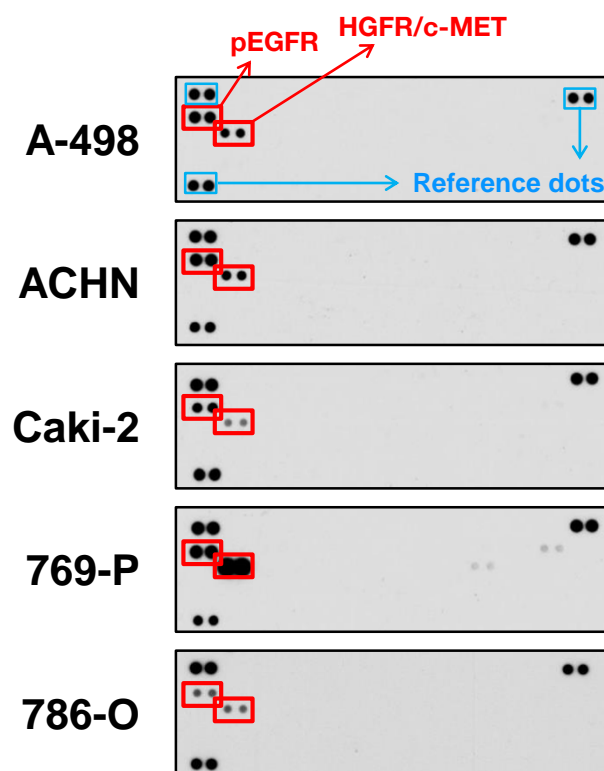

Supplementary figure 5

Supplement: Supplementary file 5 — Additional file 5: Supplementary Fig. 5. RTK activation arrays of renal cancer cell lines. The activated RTKs detected are highlighted with a red square. [file 13046_2021_2051_MOESM5_ESM.pdf]
